# Detecting Causality by Combined Use of Multiple Methods: Climate and Brain Examples

**DOI:** 10.1371/journal.pone.0158572

**Published:** 2016-07-05

**Authors:** Yoshito Hirata, José M. Amigó, Yoshiya Matsuzaka, Ryo Yokota, Hajime Mushiake, Kazuyuki Aihara

**Affiliations:** 1 Institute of Industrial Science, The University of Tokyo, 4-6-1 Komaba, Meguro-ku, Tokyo, 153–8505, Japan; 2 Centro de Investigación Operativa, Universidad Miguel Hernández, Avda. de la Universidad s/n, 03202, Elche, Spain; 3 Department of Physiology, Tohoku University School of Medicine, 2–1 Seiryo-machi Aoba-ku, Sendai, Miyagi, 980–8575, Japan; Lanzhou University of Technology, CHINA

## Abstract

Identifying causal relations from time series is the first step to understanding the behavior of complex systems. Although many methods have been proposed, few papers have applied multiple methods together to detect causal relations based on time series generated from coupled nonlinear systems with some unobserved parts. Here we propose the combined use of three methods and a majority vote to infer causality under such circumstances. Two of these methods are proposed here for the first time, and all of the three methods can be applied even if the underlying dynamics is nonlinear and there are hidden common causes. We test our methods with coupled logistic maps, coupled Rössler models, and coupled Lorenz models. In addition, we show from ice core data how the causal relations among the temperature, the CH_4_ level, and the CO_2_ level in the atmosphere changed in the last 800,000 years, a conclusion also supported by irregularly sampled data analysis. Moreover, these methods show how three regions of the brain interact with each other during the visually cued, two-choice arm reaching task. Especially, we demonstrate that this is due to bottom up influences at the beginning of the task, while there exist mutual influences between the posterior medial prefrontal cortex and the presupplementary motor area. Based on our results, we conclude that identifying causality with an appropriate ensemble of multiple methods ensures the validity of the obtained results more firmly.

## Introduction

Complex networks are ubiquitous in the real world, the brain and the earth’s climate being two typical examples that we are going to study in this paper. From the viewpoint of complex networks, the brain is a small world network, i.e., most of the brain areas are connected within a short distance effectively [1], while the climate on the earth is characterized by spatially connected regions with a high betweeness centrality, which implies the existence of large dynamical information flows actually conveyed by ocean surface currents [2]. To obtain a deeper understanding of such complex networks, a correct identification of the interactions among their sub-systems is necessary. Therefore, we refer to such interactions as directional couplings to indicate clearly that one sub-system influences another. Thus, we consider here directed networks, contrary to [1] and [2], where only undirected networks are considered.

The detection of directional couplings from time series data has been investigated extensively in the past. The most common approach is the Granger causality test [3], which evaluates the predictability when some components are hidden. In general, this test assumes a linear stochastic system [3]. Other methods focus particularly on how to deal with the nonlinearity of a system [4–17]. As it turns out, each method has its drawbacks, the major obstacle being typically a hidden common cause that affects the observed sub-systems [14].

In this report, we combine three methods for identifying directional couplings in order to overcome their drawbacks and thus achieve an accurate understanding of the interactions among the sub-systems of a system. For this purpose, we are going to propose first one already known method and then two additional new methods, all of which can be used even in the presence of hidden common causes. Finally, the overall performance of the three methods will be compared with the method by Sugihara *et al*. [16] (convergent cross mapping), which is also claimed to be applicable under such circumstances.

## Methods

**Fig 1:**
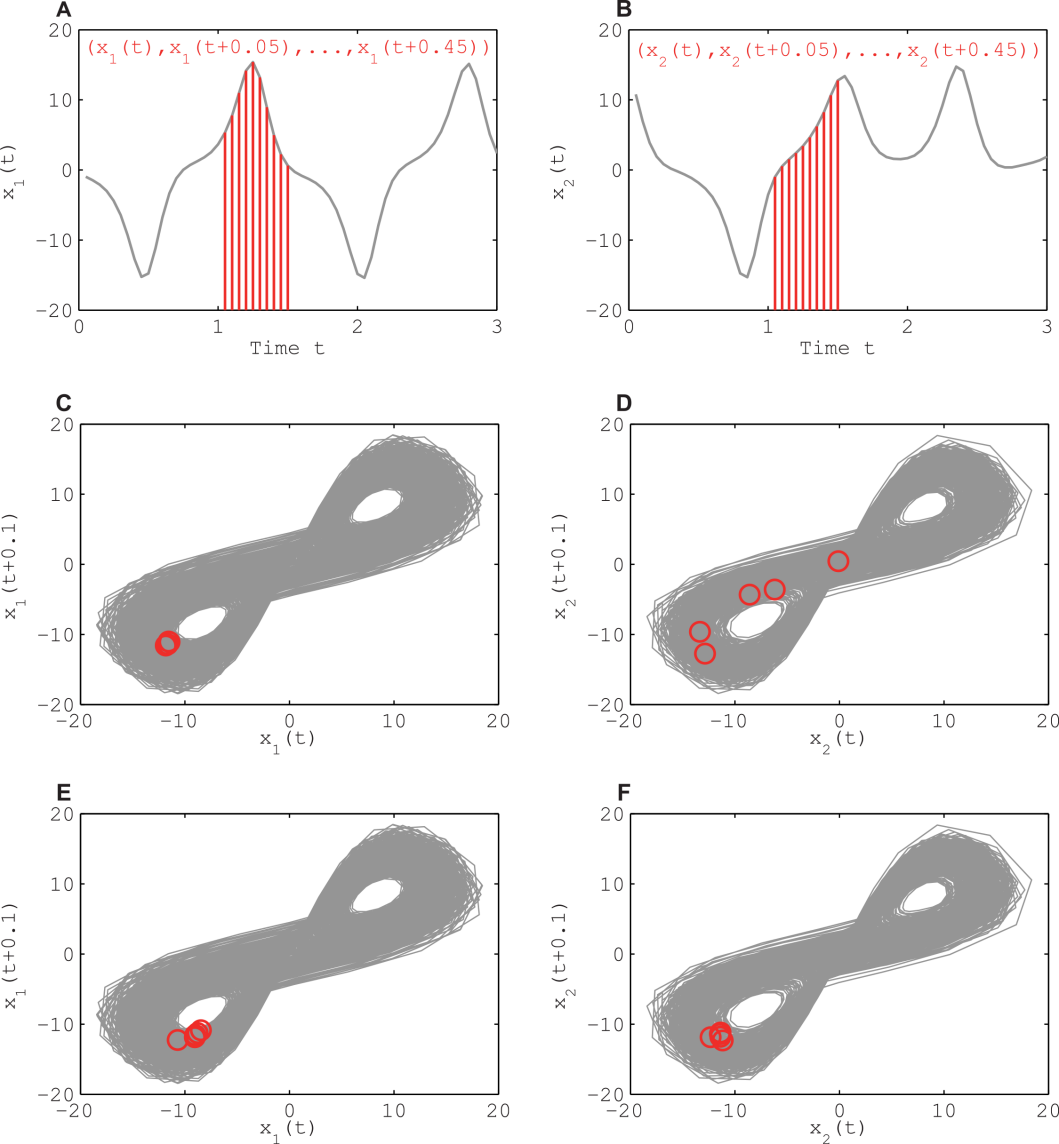
Schematic diagram of a forced system with delay coordinates. Here the Lorenz model containing the coordinate *x*_1_ drives another Lorenz model containing the coordinate *x*_2_. Panels A and B show time series of *x*_1_ and *x*_2_ as well as examples of 10-dimensional delay coordinates, respectively. Take 10 neighbors (red circles) of the driver in the 10 dimensional space spanned by the delay coordinates (panel C) to see that the corresponding time points (red circles) are scattered in the reconstructed driven system (panel D). But, when the initial 10 neighbors (red circles) are taken in the driven system (panel F), the corresponding time points (red circles) remain close to each other in the delay coordinates of the driver system (panel E).

Our first method employs the inclusive relations of recurrence plots proposed by Hirata and Aihara [14]. To the best of our knowledge, this was the first method able to identify directional couplings under the existence of unobservable common driving forces for nonlinear dynamics. The fundamental input is the delay coordinates of a forced system [18], which allows reconstruction of the joint state of the forced system and the driving forces. It turns out that neighboring points in the reconstructed driving system (Fig 1C) need not be close to each other in the reconstructed driven system (see Fig 1D), while neighbors in the reconstructed driven system (Fig 1F) remain close to each other in the reconstructed driving system (see Fig 1E), at least if the dimension is sufficiently large (see Fig 1; this example was generated using the coupled Lorenz models discussed below). Hirata and Aihara [14] implemented this principle by means of recurrence plots [19, 20] to test whether hypothetical directional couplings can be refuted. Later, Sugihara *et al*. [16] used the same principle but adopted a different implementation to identify directional couplings in such a situation. The method of Sugihara *et al*. [16] is called convergent cross mapping. Therefore, the reconstruction of the state space with delay coordinates is the key for identifying directional couplings.

**Fig 2:**
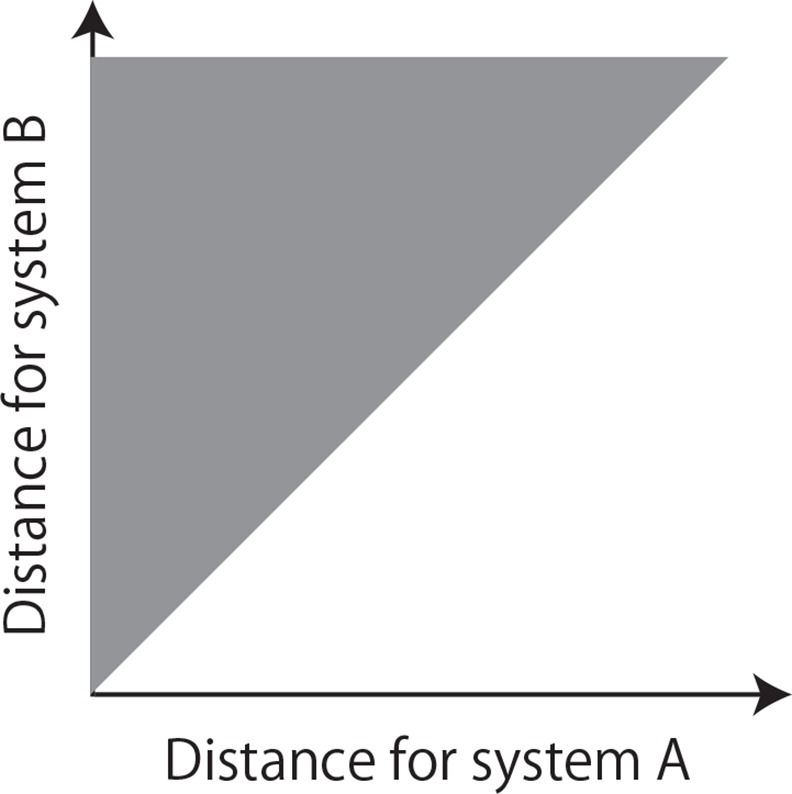
Schematic diagram of a method for identifying directional couplings using the joint distribution of distances of two sub-systems.

In our second method we employ again the delay coordinates of a forced system [18] to identify directional couplings, this time via the joint distribution of distances for two sub-systems. This implementation differs, therefore, from the implementations of Hirata and Aihara [14] and Sugihara *et al*. [16]. If there is a directional coupling from system *A* to system *B*, the delay coordinates of system *B* reconstruct the joint states of system *B* and system *A*, whereas the delay coordinates of system *A* reconstruct only the states of system *A* [21, 22]. Therefore, if two points are close to each other in terms of the delay coordinates of system *B*, they are also neighbors in terms of the delay coordinates of system *A*. It follows that the joint distribution of normalized distances for the two systems in terms of their delay coordinates is ideally located only in a triangular region, as shown in Fig 2. We quantify this characteristic to test the directional coupling from system *A* to system *B* (see Methods A in S1 File for further details).

Our third method is based on the transfer entropy [6] evaluated with permutations [23, 24]. If system *A* drives system *B*, there is an information flow from system *A* to system *B*. We quantify this information by the mutual information of transcripts [24], which are permutations obtained algebraically from pairs of permutations. To judge whether this information is significantly large, we use symbolic dynamics on permutations constructed empirically from the observed data and generate 100 surrogate series of permutations for system *B*. If the original information transferred from system *A* to system *B* is within the top *q*_*r*_% of the information transferred from system *A* to the surrogate series for system *B*, we conclude that there is a directional coupling from system *A* to system *B* (see Methods B in S1 File for further details of our implementation).

We combine the three above-mentioned methods as follows. When we apply these three methods and at least two of them detect directional couplings from system *A* to system *B*, we say that overall directional coupling is detected from system *A* to system *B*. Furthermore, we calculate the p-value using the binomial distribution where the number of independent experiments is the total number of tests and the probability of rejection of each test is *q*_*r*_, except for the case of the monkey experiment discussed later.

## Results

### Toy examples

**Fig 3:**
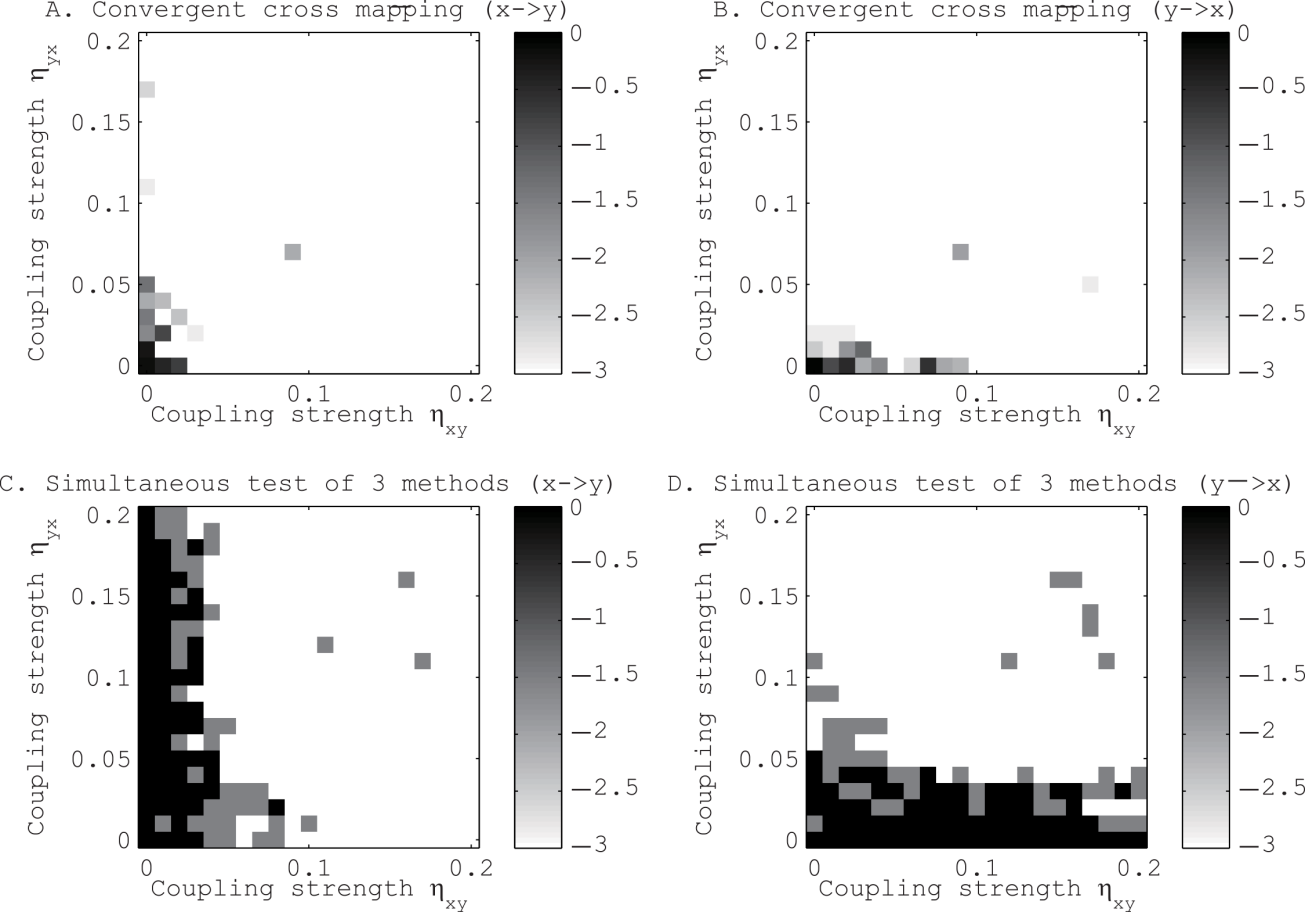
Tests for the existence of directional coupling between mutually coupled logistic maps, *x* and *y*. Panels A and B show the results for the method proposed by Sugihara *et al*. [16], and panels C and D show the results for our method. Panels A and C refer to the direction from x to y, and panels B and D refer to the direction from y to x. The gray scales show the p-values in the logarithmic scale of base 10.

Before applying our approach to real data, we tested its performance against the convergent cross mapping [16] with toy models. In all the toy models, we set *q*_*r*_ = 0.01. The first toy model consists of two logistic maps [25] coupled mutually (see Methods E in S1 File for the detailed setups). We found that our method tends to identify the directional couplings more accurately than the method proposed by Sugihara *et al*. [16] (see Fig 3). In particular, increasing the coupling strength of one directional coupling affected the identification of the other directional coupling in the convergent cross mapping [16], but not in our approach.

**Fig 4:**
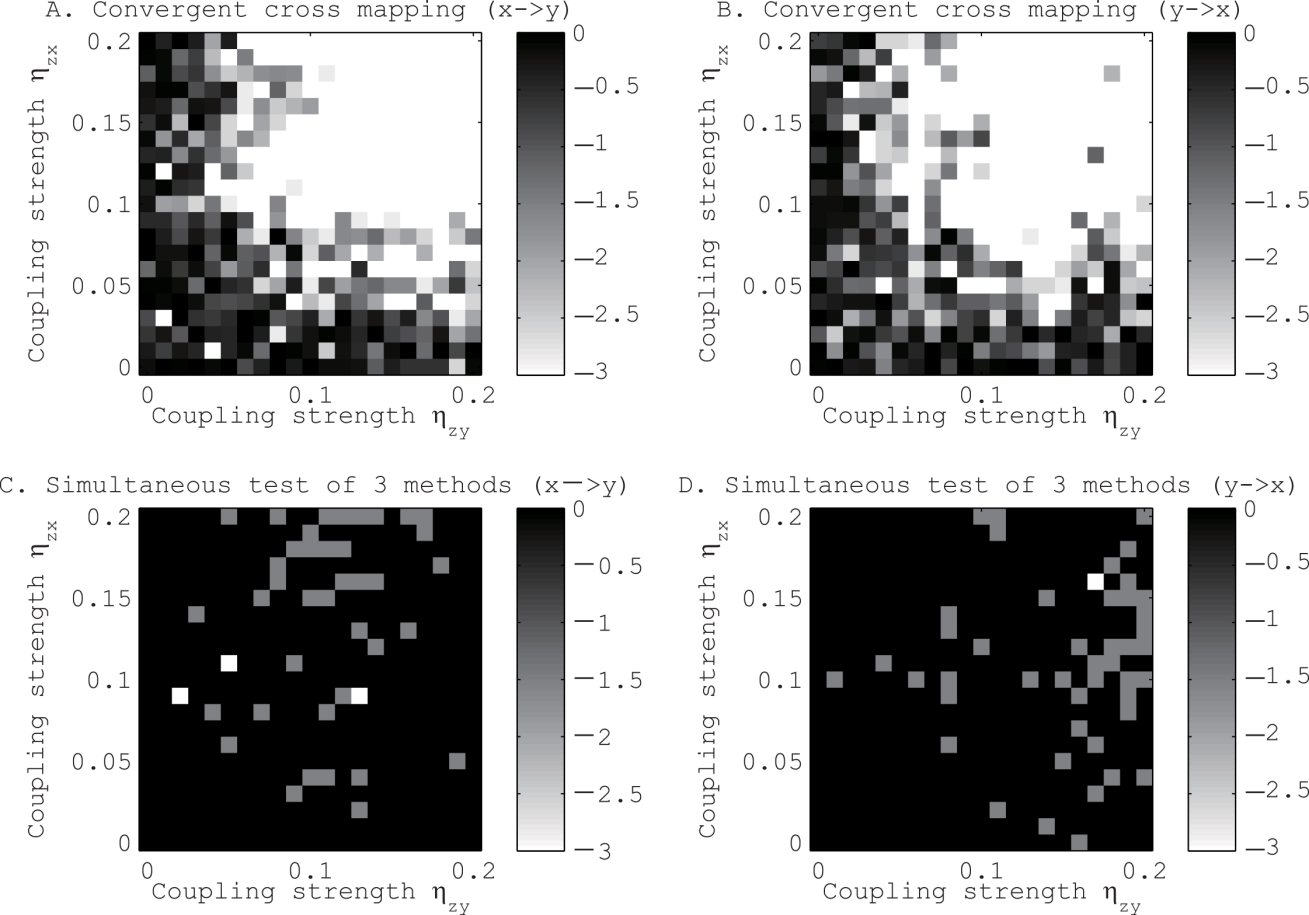
Tests for the existence of directional coupling between logistic maps driven by another logistic map. Panels A and C are for the coupling from x to y. Panels B and D are for the coupling from y to x. See the caption of Fig 3 to interpret the results.

The second toy model consists of two uncoupled logistic maps driven by another logistic map (see Fig 4). Although the convergent cross mapping [16] was affected to a considerable extent by the couplings with the third logistic map, the combined application of our three methods was not affected significantly by this common driver.

**Fig 5:**
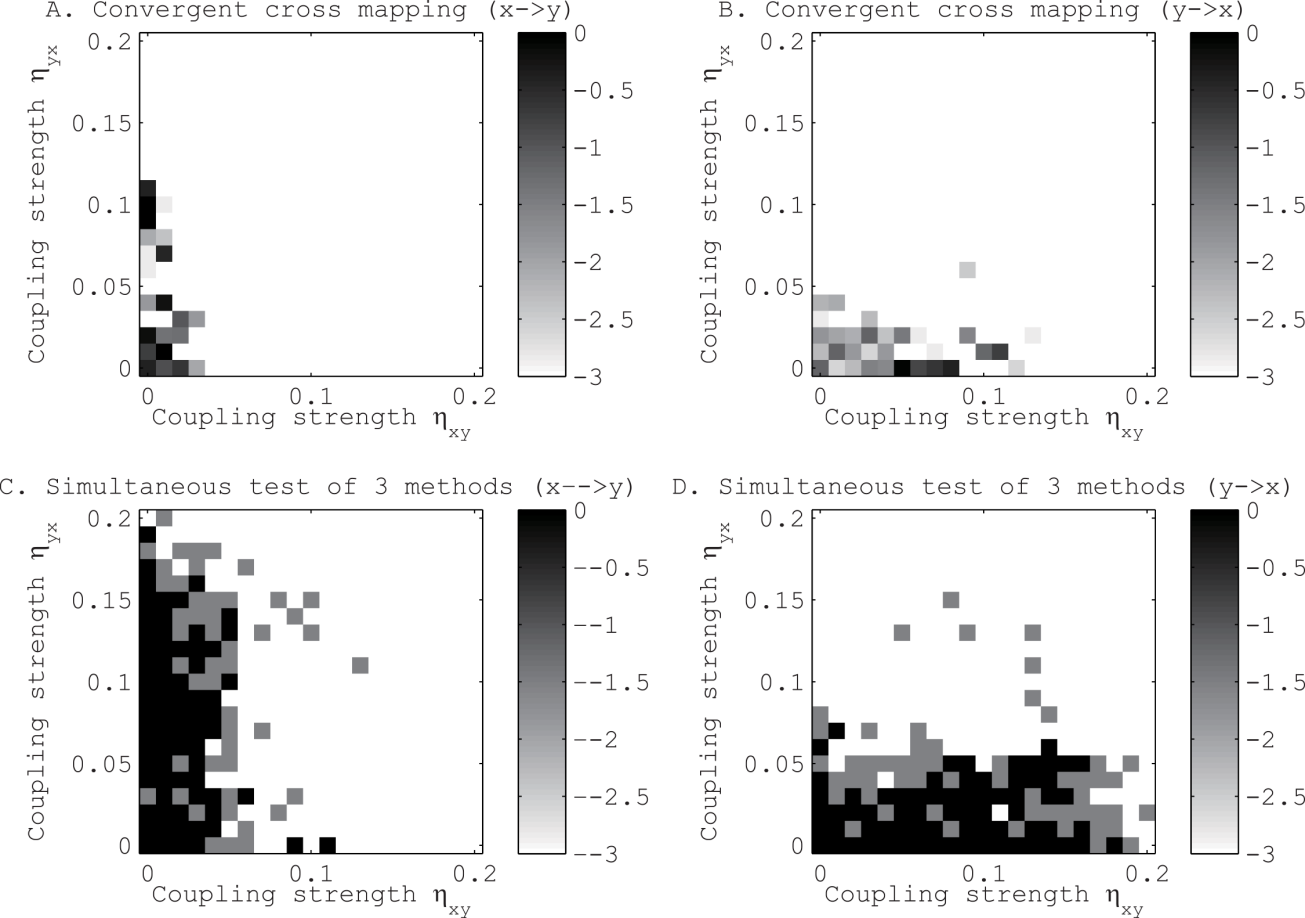
Tests for the existence of directional coupling between two mutually coupled logistic maps, *x* and *y*, driven by another logistic map, *z*. Panels A and C refer to the direction from x to y, and panels B and D refer to the direction from y to x. A and B show the results with the method proposed by Sugihara *et al*. [16], and C and D show the results with our method. See the caption of Fig 3 to interpret the results.

The third toy model consists of two mutually coupled logistic maps driven by another logistic map (see Methods G in S1 File for further details). The results shown in Fig 5 indicate that the directional couplings were almost accurately identified by our method, whereas the convergent cross mapping [16] did not work well, i.e., false positives were detected when the opposite directional couplings were strong.

**Fig 6:**
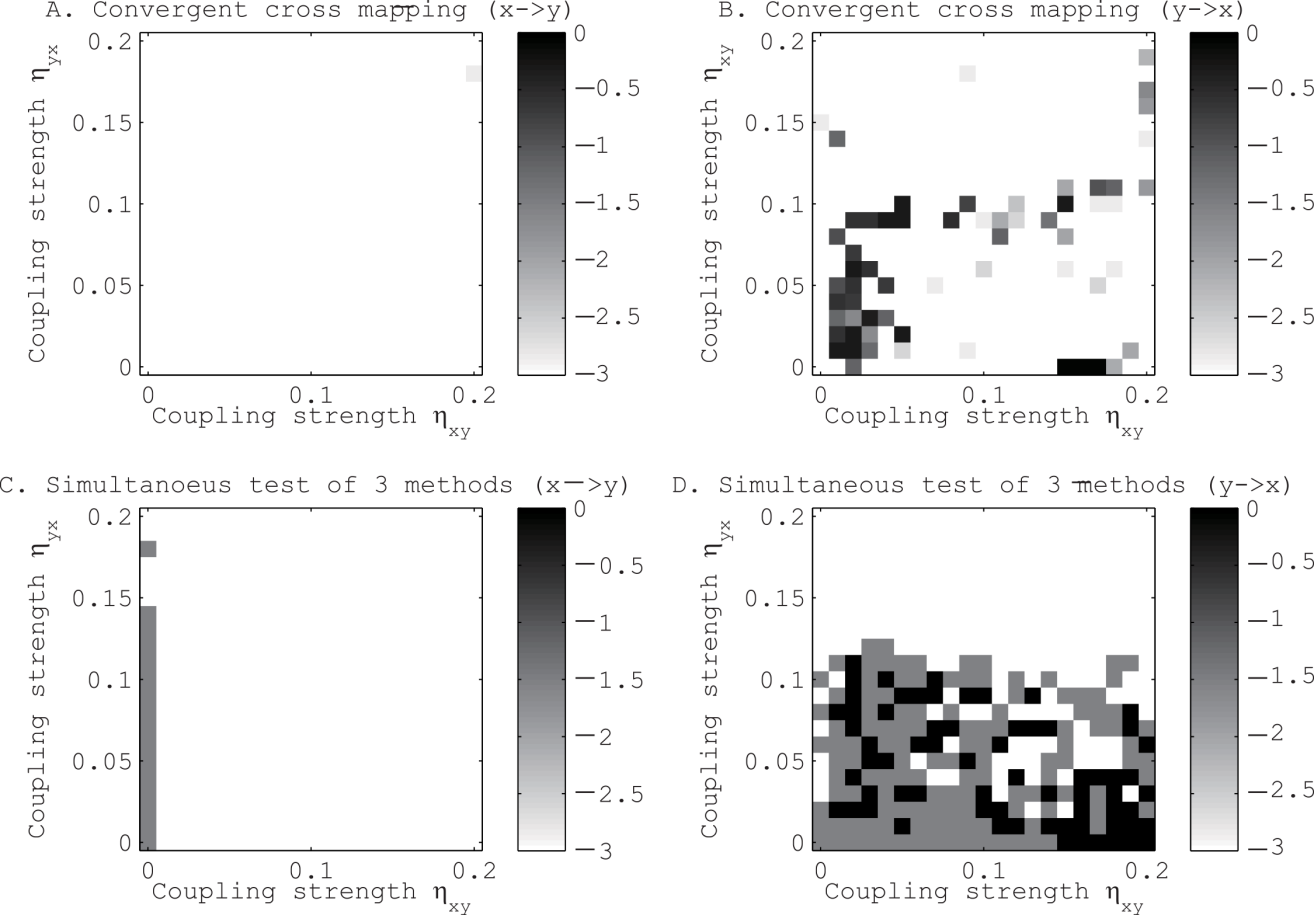
Tests for the existence of directional coupling between logistic maps mutually coupled through nonlinear effects. See the caption of Fig 3 to interpret the results.

The fourth toy model is composed of two logistic maps coupled with nonlinear terms (see Methods H in S1 File for further details). Even in this case, the results of the proposed approach were more accurate than those of the convergent cross mapping [16] (Fig 6).

The four toy models described above are examples based on maps. As the fifth example we considered the Rössler models [26] coupled as in the test conducted by Hirata and Aihara [14] (see Methods I in S1 File for further details). The results shown in Tables A and B in S1 File indicate that both our method and the method proposed by Sugihara *et al*. [16] could detect the directional couplings accurately in this case.

For our sixth toy example, coupled Lorenz models (see Methods J in S1 File for further details), both the proposed method and the convergent cross mapping [16] revealed the correct network structure when the length of time series was sufficiently long (see Tables C and D in S1 File).

### Climate data

**Fig 7:**
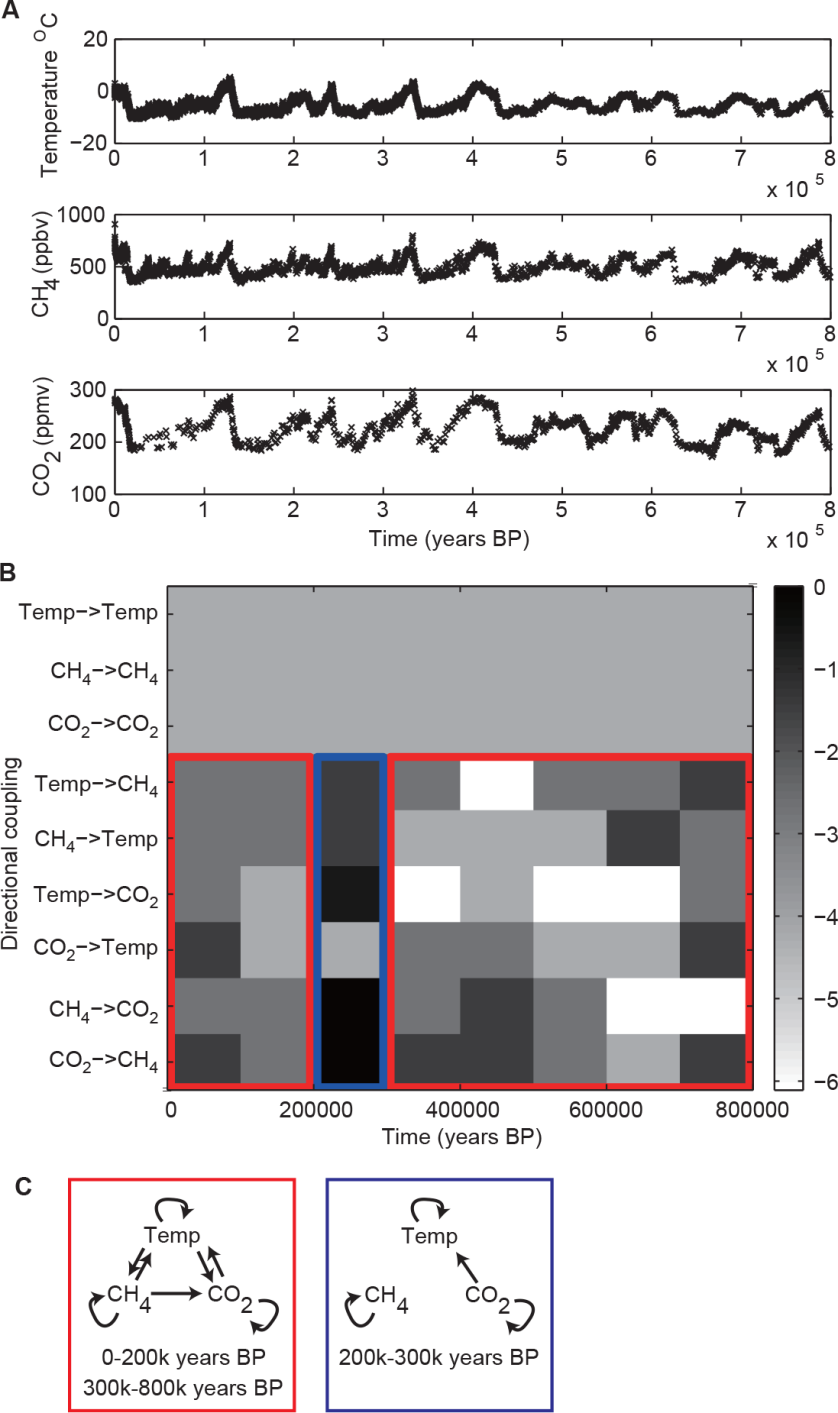
Results obtained from ice core data. In panel A, the original datasets are shown. In panel B, p-values for directional couplings are shown in a logarithmic gray scale of base 10. If the number of detections is at least 2, then the detected directional couplings are significant with an overall p-value less than 5%. In the red and blue boxes the classification results are shown. In panel C, the typical network structure for each classification is shown. The concentration of CO_2_ in the atmosphere always drove the temperature.

As for the real-world applications of our three-method approach, the first one was to (irregularly sampled) ice core data of the atmospheric CO_2_ concentration [27], CH_4_ concentration [28], and temperature [29], which share common hidden causes such as solar activity and air dusts (see Text B in S1 File for additional references to the datasets). When we divided the time axis between 0 and 800,000 years before present (BP) evenly into 8 intervals, as shown in Fig 7A, we found that the network structure changed with time. We verified that the results obtained from interpolations matched well with those obtained using raw, irregularly sampled data (the correlation coefficient of the p-values for the directional couplings was 0.26, its p-value being 0.027), and thus we obtained the combined results shown in Fig 7B. We applied the k-means algorithm [30] 100 times with different initial conditions on the temporal network topologies described by the number of rejections for each potential coupling and time segment. By choosing the classifications with the smallest classification error, we finally classified the network structure into 2 types (see Fig 7C). In the first type, the CO_2_ concentration and the temperature drive each other, the CH_4_ concentration and the temperature drive each other too, and the CH_4_ concentration drives the CO_2_ concentration only. In the second type, the CO_2_ concentration drives the temperature. Therefore, in either type, the temperature is driven by the CO_2_ concentration in the atmosphere. As shown in Fig 7C, the second type of connectivity held in the period 200k-300k years BP, while the first type did in the remaining two periods.

### Brain data

**Fig 8:**
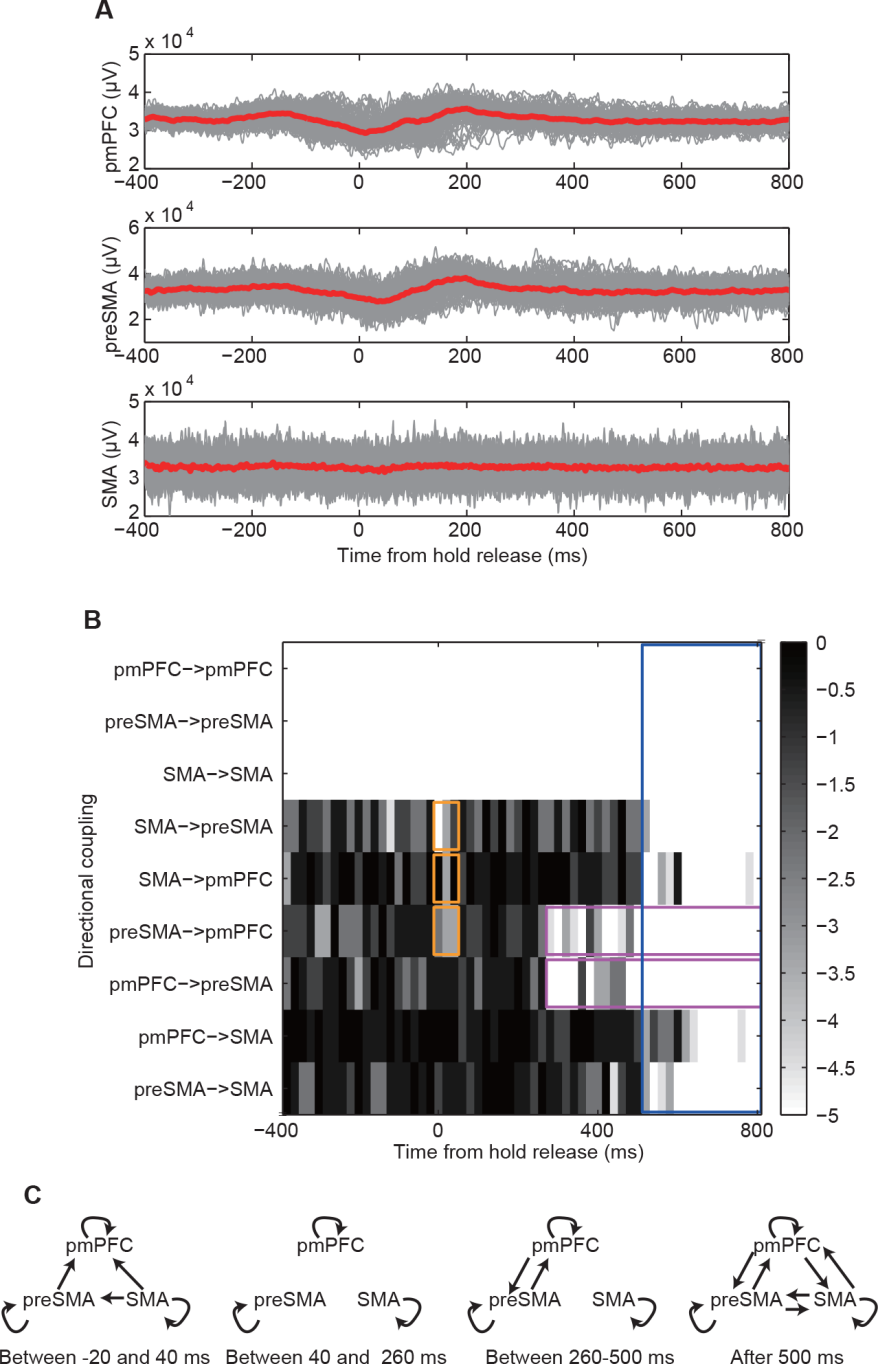
Changes in directional couplings during the visually cued, two-choice arm-reaching task. Panel A is an example of the original time series. In each sub-panel, the gray lines show time courses of individual trials and the red thick line shows their mean. Panel B shows the p-values for the corresponding directional couplings in the corresponding time intervals. The orange, magenta, and blue rectangles highlight the pairs of time windows and directional couplings for which the detected directional couplings are strong. Panel C shows the typical network structure for the corresponding time.

Furthermore, we also applied our framework to elucidate the internal brain process underlying the generation of monkey’s reactions in a visually cued, two-choice arm-reaching task [31]. The animals were treated and cared for in accordance with the National Institute of Health guidelines and the institutional guidelines for animal experiments of Tohoku University. Two of us had recorded, in Ref. [31], local field potentials from the three cortical areas, the posterior medial prefrontal cortex (pmPFC), the presupplementary motor area (preSMA), and the supplementary motor area (SMA). Here we analyzed the datasets for characterizing the temporal dynamics by their directional couplings. An example of time series data is shown in Fig 8A.

The analysis revealed that there were no causative influences with significance levels of less than 0.0001 between -400 and -20 ms before the hold release. Then, the bottom-up causative influences emerged from SMA to preSMA, from preSMA to pmPFC, and from SMA to pmPFC (beginning 20 ms before and ending 40 ms after the movement onset, the orange rectangle of Fig 8B), followed by enhanced mutual intermittent influences between pmPFC and preSMA (between 260 and 500 ms after the movement onset, the magenta rectangles of Fig 8B). Finally (500ms and later), strong and mutual influences developed among the three regions (pmPFC, preSMA and SMA, the blue rectangle of Fig 8B). In short, several regions are connected with one another when a monkey makes a decision: at the beginning, the bottom up signals elicit the decision-making process (see Fig 8C), and in the middle time period, the communication between pmPFC and preSMA seems to play a central role during the decision-making process (see Fig 8C). These observations could serve as the first crucial step in understanding how a monkey makes a decision (see Text C in S1 File for further discussion of these results).

## Discussion

In general, a set of multiple pieces of evidence provides a stronger argument than a single piece, thereby enriching our viewpoints. This way of using multiple pieces of evidence can be regarded as a restatement of the law of large numbers [32]. In recent years, this type of approach has gained popularity in the field of systems biology [33]. In the field of nonlinear time series analysis, to the best of our knowledge this approach was first adopted in [34] to argue that the behavior of a squid giant axon is chaotic.

In this paper, we carefully selected our ensemble of methods for identifying directional couplings so that each method can deal with (i) nonlinear dynamics, (ii) mutual couplings, and (iii) hidden common causes (iv) without assuming a specific model. Therefore, although there are many excellent linear methods [35–40] that can identify directional couplings, some of which can be applied even when there are some hidden components [37–40], and there are some methods that can identify which coupling direction is more dominant even when there are some hidden components [41, 42], we did not include these methods in our ensemble because none of these methods simultaneously satisfy the above four conditions. Thus, if one develops more qualified methods to identify directional couplings and use them simultaneously, the reliability of the results can improve.

S1–S4 Figs show that each of the three methods provided us independent results to some extent, which are important for the proposed framework to work appropriately. In particular, S1 and S3 Figs show that one of the three methods sometimes fails to detect directional couplings, while the failures were covered by the other two. Importantly, we observed a tendency that each of the three methods is likely to produce a false negative but not a false positive.

When we had shorter time series of network activities with too weak or strong coupling strengths, it became difficult for the proposed method to detect a directional coupling (S5–S10 Figs). The main reason is that the method of recurrence plots and the method using transfer entropy could not detect strong couplings. However, since the results are likely to be false negatives, detected couplings might be assumed to exist. This observation means that we might weaken the significance level and set the total significance level to 0.05. If we set the total significance level weaker, the above problems in S5 and S9 Figs can be avoided.

When we added 10% observational noise, the proposed framework still keeps working appropriately to some extent (S11–S16 Figs), although we observed a parameter region in the middle of each of panels C and D of S11 Fig where the p-value is still 5% significant but larger than 1%. Since this problem commonly appeared in the method using recurrence plots, the method of joint distribution of distances, and the convergent cross mapping, the problem might be related to delay coordinates. However, the observations of panels C and D of S11 Fig are related to false negatives but not false positives, hence we may assume that detected couplings are likely to exist, while there might be a chance that we miss some of directional couplings. Thus, these examples show that the chosen ensemble of the three methods is a nice combination of methods.

As we did in the Introduction, we define a directional coupling as an effect or influence that a subsystem has on another; For the ice core data, the present study indicated that the causal relationship between the CO_2_ concentration and the temperature remained constant: i.e. the CO_2_ concentration consistently drove the temperature over the time period we analyzed (Fig 7). This finding would support the hypothesis that the atmospheric CO_2_ concentration is the cause of the current global warming.

On the other hand, our results uncovered the dynamic change in the causal influences between the cortical areas (preSMA and pmPFC) during task performance (Fig 8): i.e. the bottom up influences were dominant in the beginning of the task, while pmPFC and preSMA exerted reciprocal influences at the middle of the task. Such dynamic modulation of network structure would enable the flexible switching of function of the neural network [43, 44].

The most important point is that these influences could not be revealed solely by observing time series and/or calculating the correlation coefficient between a pair of their components.

Our results in Fig 7 are consistent with those of [45] because we also found both directional couplings between the temperature and the CO_2_ concentration except for the period 200,000–300,000 years before. Our results are also consistent, to some extent, with the ones obtained by convergent cross mapping in [46]. On one hand, we detected as in [46] bi-directional couplings between the temperature and CO_2_ level, as well as between the temperature and CH_4_ level. On the other hand, we found a uni-directional coupling from CH_4_ to CO_2_, while the authors of [46] found a bi-directional coupling between CH_4_ and CO_2_.

In Fig 8, we detected directional couplings from SMA to preSMA and from preSMA to pmPFC between 0 and 20ms from the hold release. The latter directional coupling might be through preSMA in comparison with the previous electrophysiological studies [47, 48]. Whether directional couplings between pmPFC and SMA should be direct or indirect must be investigated in the future by methods such as those in [49] and [50].

When we analyzed the brain data, we did not use the k-means algorithm because the beginning of the task is a kind of a singular point and cannot be represented well by a set of centroids used in the k-means algorithm.

To conclude, the two key points of our framework can be summarized as follows. First, we use three methods for identifying directional couplings, all of which can be used for potentially mutually coupled nonlinear systems whose families of equations are not known even in the presence of hidden common causes. Second, these methods are applied concurrently in order to gain a better understanding of the network structure. In case that the results are not unanimous, the question of whether or not a directional coupling exists is settled by the majority vote. This enhances the statistical significance of the acceptance or rejection of a causal relation. Thus, as stated in the caption of Table B in S1 File, a significance level of 0.01 for each test in the system composed of five coupled Rössler oscillators is lowered to 3.0 × 10^−4^ when two tests resulted in rejection. As shown by the examples of climate and brain data presented above, our framework provides insights into interactions among sub-systems in scientific, technological, and social contexts.

## Supporting Information

S1 Fig Performance of each of the three methods for Fig 3. Panels A i), A ii), and A iii) correspond to the coupling direction from x to y, while panels B i), B ii), and B iii) correspond to the coupling direction from y to x. Panels A i) and B i) are for the method using recurrence plots. Panels A ii) and B ii) are for the method of joint distribution of distances. Panels A iii) and B iii) are for the method of transfer entropy. See the caption of Fig 3 to interpret the results for each panel.
(EPS)

S2 Fig Performance of each of the three methods for Fig 4. See the caption of S1 Fig to interpret the results.
(EPS)

S3 Fig Performance of each of the three methods for Fig 5. See the caption of S1 Fig to interpret the results.
(EPS)

S4 Fig Performance of each of the three methods for Fig 6. See the caption of S1 Fig to interpret the results.
(EPS)

S5 Fig Tests for the existence of directional coupling between mutually coupled logistic maps, x and y, the case where the length of time series is 250. See the caption of Fig 3 to interpret the results.
(EPS)

S6 Fig Performance for each of the three methods for Fig E. See the caption of S1 Fig to interpret the results.
(EPS)

S7 Fig Tests for the existence of directional coupling between logistic maps driven by another logistic map, the case where the length of time series is 250. See the caption of Fig 3 to interpret the results.
(EPS)

S8 Fig The performance for each of the three methods for S7 Fig. See the caption of S1 Fig to interpret the results.
(EPS)

S9 Fig Tests for the existence of directional coupling between mutually coupled logistic maps, x and y driven by another, the case where the length of time series is 250. See the caption of Fig 3 to interpret the results.
(EPS)

S10 Fig The performance for each of the three methods for S9 Fig. See the caption of S1 Fig to interpret the results.
(EPS)

S11 Fig Tests for the existence of directional coupling between mutually coupled logistic maps, x and y, under 10% observational noise. See the caption of Fig 3 to interpret the results.
(EPS)

S12 Fig The performance for each of the three methods for S11 Fig. See the caption of S1 Fig. to interpret the results.
(EPS)

S13 Fig Tests for the existence of directional coupling between logistic maps driven by another logistic map, under 10% observational noise. See the caption of Fig 3 to interpret the results.
(EPS)

S14 Fig The performance for each of the three methods for S13 Fig. See the caption of S1 Fig. to interpret the results.
(EPS)

S15 Fig Tests for the existence of directional coupling between mutually coupled logistic maps, x and y driven by another logistic map, under 10% observational noise. See the caption of Fig 3 to interpret the results.
(EPS)

S16 Fig The performance for each of the three methods for S15 Fig. See the caption of S1 Fig. to interpret the results.
(EPS)

S1 File Supplementary Information including Methods, Text, and Tables A-H. (PDF)
