# Correction: Detecting Causality by Combined Use of Multiple Methods: Climate and Brain Examples

**DOI:** 10.1371/journal.pone.0160864

**Published:** 2016-08-03

**Authors:** The PLOS ONE Staff

The following information is missing from the Funding section: This study was also supported by JSPS KAKENHI Grant Number 15H05879. The publisher apologizes for this error.
