# Supplementary material for: Detecting Causality by Combined Use of Multiple Methods: Climate and Brain Examples
Source: PLoS One. 2016 Jul 5;11(7):e0158572. doi: 10.1371/journal.pone.0158572 (PMC4933387; doi:10.1371/journal.pone.0158572)
Supplement: S1 File — (PDF) [file pone.0158572.s017.pdf]

Supplementary Information:

Detecting Causality by Combined Use of Multiple Methods: Climate and Brain Examples

Yoshito Hirata, José M. Amigó, Yoshiya Matsuzaka, Ryo Yokota, Hajime Mushiake, Kazuyuki Aihara

Methods

Text

Tables A-H

Methods:

Methods A: Joint distribution of distances

Suppose that $\{x_i(t)|t=1,2,\dots,T\}$ and $\{x_j(t)|t=1,2,\dots,T\}$ are two scalar time series, whose relation is our current interest. First, we embed these time series using delay coordinates $\vec{x}_i(k)=\left(x_i(D(k-1)+1),x_i(D(k-1)+2),\dots,x_i(Dk)\right)$, and $\vec{x}_j(k)=\left(x_j(D(k-1)+1),x_j(D(k-1)+2),\dots,x_j(Dk)\right)$. Here, we used $D=10$. Second, we calculate the distances $d_i(k,l)=\|\vec{x}_i(k)-\vec{x}_i(l)\|$ and $d_j(k,l)=\left\|\vec{x}_j(k)-\vec{x}_j(l)\right\|$ for each pair of (k,l), where $0\le k,l<T/D$. Then, we normalize $\{d_i(k,l)\}$ and $\{d_j(k,l)\}$ so that the corresponding $\{\tilde{d}_i(k,l)\}$ and $\{\tilde{d}_j(k,l)\}$ become uniformly distributed between 0 and 1. When there is a coupling from system i to system j, we can expect that $\tilde{d}_i(k,l)$ is small when $\tilde{d}_j(k,l)$ is small. To test this tendency, we divide the interval [0,1] for the distances $\tilde{d}_i(k,l)$ evenly into B bins, and for each bin b $(1\le b\le B)$, we obtain the minimum value $e_j(b)=\min_{\frac{b-1}{B}\le\tilde{d}_i(k,l)<\frac{b}{B}}\{\tilde{d}_j(k,l)\}$. We declare that there is a directional coupling from system i to system j if we reject the null hypothesis that the mean of $\{e_j(b+c)-e_j(b)|b=1,2,\dots,B-c\}$ is zero using the *t*-test. We set $B=20$ and

$c = 10$.

Methods B: Transfer entropy with transcripts

Transfer entropy was first proposed by Schreiber [6]. The transfer entropy from system j to system i quantifies the mutual information between $x_i(t+1)$ and $x_j(t)$ given $x_i(t)$. In its simplest expression, the transfer entropy is defined as

$$I\big(x_i(t+1); x_j(t) | x_i(t)\big) = \sum_{x_i(t+1), x_j(t), x_i(t)} \left\{ P\left(x_i(t+1), x_j(t), x_i(t)\right) \times \log_2 \frac{P\big(x_i(t+1)|x_j(t), x_i(t)\big)}{P\big(x_i(t+1)|x_i(t)\big)} \right\}, \qquad \text{(S1)}$$

where P denotes probability. Transfer entropy was later defined using permutations [23] in [51]. Permutations are defined by ranks of consecutive numbers. Let $x_i(t), \ldots, x_i(t+m-1)$ be m consecutive numbers. Then, sort these numbers such that we have $x_i(t+\pi_0(t,i)) < x_i\big(t+\pi_1(t,i)\big) < \cdots < x_i\big(t+\pi_{m-1}(t,i)\big)$. If $x_i\big(t+\pi_g(t,i)\big) = x_i(t+\pi_h(t,i))$ and $g < h$, we define $\pi_g(t,i) < \pi_h(t,i)$. The set of numbers $\{\pi_h\}$ defines a permutation of the numbers $\{0,1,\ldots,m-1\}$. The symbolic transfer entropy from system j to system i is then defined as [51]

$I\big(\pi(t+1,i);\pi(t,j)\big|\pi(t,i)\big) = H\big(\pi(t+1,i)\big|\pi(t,i)\big) - H\big(\pi(t+1,i)\big|\pi(t,j),\pi(t,i)\big).$

(S2)

Recently, Monetti *et al.* [24] proposed the mutual information of transcripts. A transcript is defined by an algebraic operation on permutations. Let α and β be two permutations. Then, the transcript from α to β is defined by $\beta\alpha^{-1}$. It was shown in [52] that under mild mathematical conditions, the symbolic transfer entropy, given by Eq. (S2), agrees with the following mutual information of transcripts:

$$I(\pi(t,i)\pi(t+1,i)^{-1}, \pi(t,i)\pi(t,j)^{-1}). \qquad \text{(S3)}$$

Eq. (S3) has only two variables, whereas Eq. (S2) has three variables; thus, the estimation of the coupling direction using Eq. (S3) is, in general, better than that using Eq. (S2). Therefore, we used Eq. (S3) to quantify the directional coupling from system j to system i. We need to quantify the significance level for the directional coupling. Here, we compare the value of Eq. (S3) for the original time series with the values of Eq. (S3) obtained from 100 surrogate data. These surrogate data were generated by symbolic dynamics on

permutations. First, we choose the initial permutations by considering the distribution of permutations of the original time series. Then, in each step, we choose the permutation to which we transit using the transition matrix of the original time series. The significance level q_r was obtained on the basis of the number of values of Eq. (S3) for the surrogate data that exceeded the original value of Eq. (S3). The length of the permutations was 2.

Methods C: Using recurrence plots

We used the same method as Hirata and Aihara [14], except that we fixed the embedding dimension to 10. We set the target significance level r=0.005 for the regularly sampled datasets and r=0.05 for the irregularly sampled datasets.

Methods D: Convergent cross mapping

We used the method proposed by Sugihara *et al.* [16] to compare the performance of our method. We obtained p-values with 1000 realizations of the iterative amplitude adjusted Fourier transform surrogates [53] for each case. (As for the appropriateness, it might have

been better to use twin surrogates [54, 55] to obtain the p-values because their null hypotheses have the same dynamics under different initial conditions; however, we followed the approach of the original paper [16].) We set the embedding dimension to 10 as in [14].

Methods E: Two logistic maps coupled mutually (first toy model)

We define two mutually coupled logistic maps as follows:

$$\mathrm{x}(t+1) = \left(1-\eta_{yx}\right)\left(3.8x(t)\left(1-x(t)\right)\right) + \eta_{yx}\left(3.81y(t)\left(1-y(t)\right)\right), \tag{S4}$$

$$\mathrm{y}(\mathrm{t}+1) = \left(1-\eta_{xy}\right)\left(3.81y(t)\left(1-y(t)\right)\right) + \eta_{xy}\left(3.8x(t)\left(1-x(t)\right)\right), \tag{S5}$$

where we varied η_{xy} and η_{yx} between 0 and 0.2. We generated a time series of length 1000 after ignoring the initial transient. Then, we evaluated whether there are directional couplings between x and y.

Methods F: Logistic maps driven by another logistic map (second toy model)

We define two uncoupled logistic maps driven by another logistic map as follows:

$$x(t+1) = (1-\eta_{zx})\left(3.8x(t)\big(1-x(t)\big)\right) + \eta_{zx}\left(3.82z(t)\big(1-z(t)\big)\right), \quad \text{(S6)}$$

$$y(t+1) = \big(1-\eta_{zy}\big)\left(3.81y(t)\big(1-y(t)\big)\right) + \eta_{zy}\left(3.82z(t)\big(1-z(t)\big)\right), \quad \text{(S7)}$$

$$z(t+1) = 3.82z(t)\big(1-z(t)\big), \quad \text{(S8)}$$

where we varied η_{zx} and η_{zy} between 0 and 0.2. For each pair of η_{zx} and η_{zy}, we generated a time series of length 1000 after ignoring the initial transient. Then, we evaluated whether there are directional couplings between x and y.

Methods G: Two mutually coupled logistic maps driven by another logistic map (third toy model)

We define two mutually coupled logistic maps driven by another logistic map as follows:

$$x(t+1) = \big(1-\eta_{yx}-\eta_{zx}\big)\left(3.8x(t)\big(1-x(t)\big)\right) + \eta_{yx}\big(3.81y(t)(1-y(t))\big) + \eta_{zx}\left(3.82z(t)\big(1-z(t)\big)\right), \quad \text{(S9)}$$

$$y(t+1) = \big(1-\eta_{xy}-\eta_{zy}\big)\left(3.81y(t)\big(1-y(t)\big)\right) + \eta_{xy}\big(3.8x(t)(1-x(t))\big) + \eta_{zy}\left(3.82z(t)\big(1-z(t)\big)\right), \quad \text{(S10)}$$

$$z(t+1) = 3.82z(t)\big(1-z(t)\big), \quad \text{(S11)}$$

where we fixed $\eta_{zx} = \eta_{zy} = 0.05$ and varied η_{xy} and η_{yx} between 0 and 0.2. For each quadruplet of η_{zx}, η_{zy}, η_{xy}, and η_{yx}, we generated a time series of length 1000 after ignoring the initial transient. Then, we evaluated whether there are directional couplings between x and y.

Methods H: Mutually coupled logistic maps with nonlinear couplings (fourth toy model)

Similarly to Sugihara *et al.* [16], we consider the following model to evaluate the effects of nonlinear couplings:

$$\mathrm{x}(t+1) = x(t)\big(3.8 - 3.8x(t) - \eta_{yx}y(t)\big), \tag{S12}$$

$$\mathrm{y(t+1) = y(t)}\big(3.5 - 3.5y(t) - \eta_{xy}x(t)\big), \tag{S13}$$

where we varied η_{yx} and η_{xy} between 0 and 0.2. For each set of parameters, we generated a time series of length 1000, after ignoring the initial transient.

Methods I: Coupled Rössler models (fifth toy model)

As in the case of Hirata and Aihara [14], we couple five Rössler models [26] as follows:

$$\dot{x}_1 = -\omega_1 y_1 - z_1, \tag{S14}$$

$$\dot{y}_1 = \omega_1 x_1 + a y_1, \tag{S15}$$

$$\dot{z}_1 = b + z_1(x_1 - c), \tag{S16}$$

$$\dot{x}_2 = -\omega_2 y_2 - z_2 + \epsilon(x_1 - x_2), \tag{S17}$$

$$\dot{y}_2 = \omega_2 x_2 + a y_2, \tag{S18}$$

$$\dot{z}_2 = b + z_2(x_2 - c), \tag{S19}$$

$$\dot{x}_3 = -\omega_3 y_3 - z_3 + \epsilon(x_1 - x_3), \tag{S20}$$

$$\dot{y}_3 = \omega_3 x_3 + a y_3, \tag{S21}$$

$$\dot{z}_3 = b + z_3(x_3 - c), \tag{S22}$$

$$\dot{x}_4 = -\omega_4 y_4 - z_4 + \epsilon(x_5 - x_4), \tag{S23}$$

$$\dot{y}_4 = \omega_4 x_4 + a y_4, \tag{S24}$$

$$\dot{z}_4 = b + z_4(x_4 - c), \tag{S25}$$

$$\dot{x}_5 = -\omega_5 y_5 - z_5 + \epsilon(x_4 - x_5), \quad \text{(S26)}$$

$$\dot{y}_5 = \omega_5 x_5 + a y_5, \quad \text{(S27)}$$

$$\dot{z}_5 = b + z_5(x_5 - c), \quad \text{(S28)}$$

where we set $a = 0.15$, $b = 0.2$, $c = 10$, $\omega_1 = 1.05$, $\omega_2 = 1.03$, $\omega_3 = 1.01$, $\omega_4 = 0.99$, $\omega_5 = 0.97$, and $\epsilon = 0.05$. Therefore, the first oscillator drives the second and third oscillators, and the fourth and fifth oscillators are mutually coupled. After discarding the initial transient, we observed x_1, x_2, x_3, x_4, and x_5 every unit time until a time series of length 2000 was obtained.

Methods J: Coupled Lorenz models (The sixth toy model)

We couple 5 Lorenz models as follows:

$$\dot{x}_1 = -s(x_1 - y_1), \quad \text{(S29)}$$

$$\dot{y}_1 = -x_1 z_1 - g x_1 - y_1, \quad \text{(S30)}$$

$$\dot{z}_1 = x_1 y_1 - b_1 z_1, \quad \text{(S31)}$$

$$\dot{x}_2 = -s(x_2 - y_2) + \epsilon(x_1 - x_2), \quad \text{(S32)}$$

$$\dot{y}_2 = -x_2 z_2 - g x_2 - y_2, \quad \text{(S33)}$$

$$\dot{z}_2 = x_2 y_2 - b_2 z_2, \quad \text{(S34)}$$

$$\dot{x}_3 = -s(x_3 - y_3) + \epsilon(x_1 - x_3), \quad \text{(S35)}$$

$$\dot{y}_3 = -x_3 z_3 - g x_3 - y_3, \quad \text{(S36)}$$

$$\dot{z}_3 = x_3 y_3 - b_3 z_3, \quad \text{(S37)}$$

$$\dot{x}_4 = -s(x_4 - y_4) + \epsilon(x_5 - x_4), \quad \text{(S38)}$$

$$\dot{y}_4 = -x_4 z_4 - g x_4 - y_4, \quad \text{(S39)}$$

$$\dot{z}_4 = x_4 y_4 - b_4 z_4, \quad \text{(S40)}$$

$$\dot{x}_5 = -s(x_5 - y_5) + \epsilon(x_4 - x_5), \quad \text{(S41)}$$

$$\dot{y}_5 = -x_5 z_5 - g x_5 - y_5, \quad \text{(S42)}$$

$$\dot{z}_5 = x_5 y_5 - b_5 z_5, \tag{S43}$$

where we used $s = 10, g = 28, b_1 = \frac{8.0}{3}, b_2 = \frac{8.1}{3}. b_3 = \frac{8.2}{3}, b_4 = \frac{8.3}{3},\ b_5 = \frac{8.4}{3}$, and $\epsilon = 1$. In this system, the first Lorenz model drives the second and the third Lorenz models, and the fourth and fifth Lorenz models are mutually coupled. We generated time series of the x_1, x_2, x_3, x_4, x_5 coordinates every 0.05 unit time up to completing 10,000 time points.

Methods K: Irregularly sampled models

We generated irregularly sampled data from the above coupled Rössler models and coupled Lorenz models. In the case of the coupled Rössler models, we sampled each of the x_1, x_2, x_3, x_4, x_5 coordinates independently in time intervals following the uniform distribution between 1.00 and 1.50 and integrated the system over a time interval of length 10000. In the case of the coupled Lorenz models, we sampled each of x_1, x_2, x_3, x_4, and x_5 independently in time intervals following the uniform distribution between 0.05 and 0.075 and integrated the system over a time interval of length 1000.

To detect directional couplings based on irregularly sampled datasets, we can use inclusive relations of recurrence plots and joint distribution of distances. For this sake, we modified the method of dynamic time warping [56] to define a distance d between two irregularly sampled data $\{(\alpha_1(j), \tau_1(j)) \in \Re^2 | j = 1,2, \dots, J\}$ and $\{(\alpha_2(k), \tau_2(k)) \in \Re^2 | k = 1,2, \dots, K\}$ in the following way. Consider a $(J+1) \times (K+1)$ matrix D. First set $D(1,1) = 0$, $D(1,k) = 0$ for $k = \{2,3, \dots, K+1\}$ and $D(j,1) = 0$ for $j = \{2,3, \dots, J+1\}$. Then, calculate $D(j+1, k+1)$ recursively as follows:

$$D(j+1, k+1) = \lambda_\alpha |\alpha_1(j) - \alpha_2(k)| + \lambda_\tau |\tau_1(j) - \tau_2(k)| + \min \{D(j,k), D(j+1,k), D(j,k+1)\}. \qquad \text{(S44)}$$

Then, define a distance between the two irregularly sampled data above by

$$d = \frac{D(J+1, K+1)}{\max \{J, K\}}. \qquad \text{(S45)}$$

We picked a time window and slid it along the time axis repeatedly by its size to find out the total number of (non-overlapping) positions of the time window. For each pair of positions of the time window, we defined their distance as in Eq. (S45). By considering the

time scales of the dynamics, we set the size of the time window to 20 unit times for the coupled Rössler models and 1 unit time for the coupled Lorenz models. For the ice core data, we chose 10,000 years for the size of the time window so that at least 3 time points are included in each time window. The parameter λ_τ was set to 1/(size of time window) and λ_α was set to 1/(size of time window)/(standard deviation of the corresponding observable for the whole data).

Text:

Text A: Irregularly sampled data (toy models)

We estimated the network structure from the irregularly sampled data generated from the coupled Rössler models by using the joint distribution of distances (Table E) and the inclusive relation of recurrence plots (Table F). We found that the joint distribution of distances estimated the network structure correctly, while the inclusive relation of recurrence plots had some errors in the estimation.

We also applied a similar test for the coupled Lorenz models. The results shown in Tables G and H show a similar tendency: the joint distribution of distances yielded the correct network structure, while the method based on the inclusive relation of recurrence plots made some errors.

Text B: Ice core data

The ice core data used in this study was downloaded from http://www.ncdc.noaa.gov/paleo/icecore/antarctica/domec/domec_epica_data.html. For completeness, we also cite [57-61]. Because the sampling intervals were different, we used the interpolation function *interp1* of MATLAB® to make the sampling intervals even so that there is one observation every 400 years. We set $q_r = 0.06$ so that we can maximally exploit the information obtained from each of 5 different tests (inclusive relation of recurrence plots, joint distribution of distances, and transfer entropy by permutations for the interpolated data, and inclusive relation of recurrence plots, and joint distribution of distances for the irregularly sampled original data).

In recent years, global warming has been investigated via time series analysis [62-66]. Ice core data has also been investigated using the Granger causality test [45]. However, because the earth's climate is driven by the sun, we need different methods, such as the three methods of our combined approach.

Text C: Visually cued, two-choice arm-reaching task

Two Japanese macaques were trained to perform a visually cued, two-choice spatial reaching task [31]. The monkeys were seated on a primate chair and faced a panel. The panel was equipped with two push buttons, right and left, each of which was back-illuminated by a full-color LED.

A trial began when the monkeys pressed a hold button attached to the primate chair with their right hand. After a variable waiting period of 1–1.5 s, the LED of one of the push buttons was turned on (either green or red), which served as the cue to determine the target

button (green for left, red for right). The location of the cue, which was either ipsilateral (concordant trial) or contralateral (discordant trial) to the target button, was irrelevant. The monkeys received a liquid reward for reaching toward and hitting the correct button within 1 s. Here, we analyzed the dataset of one of the two macaques because for the other, no simultaneous measurements of the three regions of the brain were recorded. In addition, we analyzed a part of the dataset where the first, second, and third electrodes were used for measuring the activity of pmPFC, preSMA, and SMA, respectively.

We divided the time axis into 20-ms bins, and in each time bin, we analyzed the datasets of multiple trials simultaneously. We set $q_r = 0.05$ so that a certain number of rejected experiments is necessary to infer a directional coupling between each pair of regions.

Additional References

51. Staniek M, Lehnertz K. Symbolic transfer entropy. Phys. Rev. Lett. 2008; 100: 158101.

52. Amigó JM, Aschenbrenner T, Bunk W, Monetti R. Dimensional reduction of conditional algebraic multi-information via transcripts. Inf. Sci. 2014; 278: 298-310.

53. Schreiber T, Schmitz A. Improved surrogate data for nonlinearity tests. Phys. Rev. Lett. 1996; 77: 635-638.

54. Thiel M, Romano MC, Kurths J, Rolfs M, Kliegl R. Twin surrogates to test for complex systems. Europhys. Lett. 2006; 75: 535-541.

55. Romano MC, Thiel M, Kurths J, Mergenthaler K, Engbert R. Hypothesis test for synchronization: Twin surrogates revisited. Chaos 2009; 19: 015108.

56. Sakoe H, Chiba S. Dynamic programing algorithm optimization for spoken word recognition. IEEE Trans. Acoust. 1978; ASSP-26: 43-49.

57. Monnin E, Indermühle A, Dällenbach A, Flückiger, J, Stauffer B, Stocker TF, et al. Atmospheric CO2 concentrations over the last glacial termination. Science 2001; 291: 112-114.

58. Petit JR, Jouzel J, Raynaud D, Barkov NI, Barnola J-M, Basile I, et al. Climate and atmospheric history of the past 420,000 years from the Vostok ice core, Antarctica. Nature 1999; 399: 429-436.

59. Pépin L, Raynaud D, Barnola J-M, Loutre MF. Hemispheric roles of climate forcings during glacial-interglacial transitions as deduced from the Vostok record and LLN-2D model experiments. J. Geophys. Res. Atmos. 2001; 106: 31885-31892.

60. Raynaud D, Barnola J-M, Souchez R, Lorrain R, Petit J-R, Duval P, et al. Palaeoclimatology: The record for marine isotopic stage 11. Nature 2005; 436: 39-40.

61. Siegenthaler U, Stocker TF, Monnin E, Lüthi D, Schwander J, Stauffer B, et al. Stable carbon cycle-climate relationship during the late Pleistocene. Science 2005; 310: 1313-1317.

62. Sun L, Wang M. Global warming and global dioxide emission: an empirical study. J. Environ. Manage. 1996; 46: 327-343.

63. Stern DI, Kaufmann RK. Econometric analysis of global climate change. Environ. Model. Softw. 1999; 14: 597-605.

64. Verdes PF. Assessing causality from multivariate time series. Phys. Rev. E 2005; 72: 026222.

65. Triacca U, Attanasio A, Pasini A. Anthropogenic global warming hypothesis: testing its robustness by Granger causality analysis. Environmetrics 2013; 24: 260-268.

66. Stern DI, Kaufmann RK. Anthropogenic and natural causes of climate change. Climate Change 2014; 122: 257-269.

Table A. Estimated network for five Rössler models coupled as described in Ref. [14] by convergent cross mapping. Symbol 0 means that the corresponding directional coupling is not detected and symbol 1 means that the corresponding directional coupling is detected with a significance level of 0.01.

From\To	1	2	3	4	5
1	1	1	1	0	0
2	0	1	0	0	0
3	0	0	1	0	0
4	0	0	0	1	1
5	0	0	0	1	1

Table B. Estimated network for five Rössler models coupled as described in Ref. [14] by the proposed framework. Shown is the number of rejections by the three methods used for the corresponding pair of supposed driver and driven sub-system. Because each test has a significance level of 0.01, the significance level is 3.0×10^{-4} when two tests output rejection.

From\To	1	2	3	4	5
1	2	2	2	0	0
2	1	2	0	0	0
3	1	0	2	1	0
4	0	0	0	2	3
5	0	0	0	3	2

Table C. Estimated network for five coupled Lorenz models by convergent cross mapping. See the caption of Table A to interpret the results.

From\To	1	2	3	4	5
1	1	1	1	0	0
2	0	1	0	0	0
3	0	1	1	1	0
4	0	0	0	1	1
5	0	0	0	1	1

Table D. Estimated network for five coupled Lorenz models by the proposed framework. See the caption of Table B to interpret the results.

From\To	1	2	3	4	5
1	2	3	2	0	0
2	0	2	0	0	0
3	0	0	2	0	0
4	0	0	0	2	3
5	0	1	0	2	2

Table E. Estimated network for five Rössler models coupled as described in Ref. [14] based on irregularly sampled data by joint distribution of distances. See the caption of Table A to interpret the results.

From\To	1	2	3	4	5
1	1	1	1	0	0
2	0	1	0	0	0
3	0	0	1	0	0
4	0	0	0	1	1
5	0	0	0	1	1

Table F. Estimated network for five Rössler models coupled as described in Ref. [14] based on irregularly sampled data by inclusive relation of recurrence plots. The significance level was 0.05. See the caption of Table A to interpret the results.

From\To	1	2	3	4	5
1	1	0	1	0	1
2	0	1	0	0	0
3	0	0	1	0	0
4	0	0	0	1	1
5	0	0	0	1	1

Table G. Estimated network for five coupled Lorenz models based on irregularly sampled data by joint distribution of distances. See the caption of Table A to interpret the results.

From\To	1	2	3	4	5
1	1	1	1	0	0
2	0	1	0	0	0
3	0	0	1	0	0
4	0	0	0	1	1
5	0	0	0	1	1

Table H. Estimated network for five Lorenz models based on irregularly sampled data by inclusive relation of recurrence plots. See the caption of Table F to interpret the results.

From\To	1	2	3	4	5
1	1	1	0	0	0
2	0	1	0	0	0
3	0	0	1	0	0
4	0	0	0	1	0
5	0	0	0	1	1
